# Sound quality, not speech recognition, explains cochlear implant-related quality of life outcomes

**DOI:** 10.1121/10.0039069

**Published:** 2025-10-01

**Authors:** Katelyn A. Berg, Hugh M. Birky, Victoria A. Sevich, Aaron C. Moberly, Terrin N. Tamati

**Affiliations:** 1 Otolaryngology—Head and Neck Surgery, Vanderbilt University Medical Center, Nashville, Tennessee 37232, USA; 2 Speech and Hearing Science, The Ohio State University, Columbus, Ohio 43210, USA

## Abstract

This study examined relationships between cochlear implant–aided speech recognition, sound quality ratings, and quality of life outcomes in 41 adult cochlear implant users. Participants completed word and sentence recognition tasks, the Speech, Spatial and Qualities (SSQ) questionnaire, and the Cochlear Implant Quality of Life (CIQOL) questionnaire. Sound quality ratings on SSQ showed a weak correlation with sentence recognition in noise but strong positive correlations with CIQOL scores. Sound quality independently predicted quality of life, explaining 32% of global score variance, while speech recognition measures showed no effects. These findings highlight sound quality as a distinct and meaningful outcome metric.

## 1. Introduction

More than 1 million people worldwide depend on cochlear implants (CIs) to hear.^1^ Yet, their sound quality experiences vary dramatically—from perceiving mostly white noise to achieving near-natural hearing.^2^ Despite this variability, clinical CI outcomes have traditionally been evaluated through isolated word and sentence recognition testing. These assessments quantify a CI user’s ability to discriminate and recognize speech in controlled conditions. While CIs generally succeed in providing at least some open-set speech recognition to users, outcomes vary considerably among recipients.^3^ Multiple interacting factors influence speech recognition outcomes, including the placement of the electrode array,^4^ the health of the auditory neural pathway,^5,6^ cognition,^7^ and device wear time.^8^ However, speech recognition measures do not strongly relate to hearing-related quality of life (QOL), suggesting that these measures may not fully capture the richness or distortion of sound perceived by CI users.

Sound quality is the listener’s perceptual reaction to a sound, encompassing multiple dimensions, such as clarity, naturalness, resonance, and distortion—the more acceptable, the greater the sound quality.^9^ The variation observed in perceived CI sound quality likely stems from how the device functions. Current CI technology has inherent limitations that affect sound quality perception, including limited spectral resolution^10^ and channel interaction between electrodes.^11,12^ Device-related factors are the primary source of variation in sound quality. However, individual factors, such as duration of device use, age at onset of hearing loss, and adaptation to the CI may also contribute to differences in subjective quality ratings.^13^ Despite these known technical limitations, the relationships between CI sound quality and other outcome measures, such as speech recognition and overall QOL, remain unexplored.

The Speech, Spatial, and Qualities (SSQ) questionnaire^14^ is part of the Minimum Speech Test Battery^15^ and provides a clinically feasible method for assessing sound quality. The SSQ assesses hearing disability across three domains—speech understanding, spatial hearing, and sound qualities. The Qualities section specifically evaluates the perceived naturalness and clarity of sounds, ability to segregate sounds, and listening effort. Previous studies have shown weak to moderate correlations between SSQ scores and speech recognition (*r* = 0.17–0.61), suggesting that sound quality ratings capture aspects of the CI listening experience that are largely independent of speech recognition abilities.^16,17^

Quality of life (QOL) is a multidimensional construct encompassing an individual’s perception of their physical, psychological, and social well-being. While general QOL captures overall life satisfaction, health-related QOL focuses on how health conditions impact daily functioning and well-being. For individuals with hearing loss, hearing-related QOL reflects the impact of auditory abilities and limitations on communication, social participation, emotional well-being, and daily activities. A meta-analysis by McRackan and colleagues showed weak correlations between speech recognition and QOL (*r* = 0.20–0.26),^18^ leaving most QOL variance unexplained. While domain-specific QOL data are limited due to using different assessment instruments across studies, prior work examining communication-related QOL has shown moderate correlations with speech recognition (*r* = 0.55–0.61).^18^ However, to our knowledge, no study to date has investigated the relationship between domain-specific QOL ratings and perceived CI sound quality ratings.

While previous studies have examined bivariate relationships between speech recognition, CI sound quality, and hearing-related QOL, the complex interplay between these outcomes likely involves multiple interacting factors that simple correlational analyses cannot capture. More sophisticated analytical approaches are needed to understand how these variables work together to influence CI user outcomes and to identify which factors most strongly predict QOL. Such analyses could identify important relationships hidden by simple correlations, potentially leading to more targeted clinical interventions to guide counseling, programming, and rehabilitation approaches.

Therefore, this study examined the relationships between CI-aided speech recognition (word and sentence recognition in quiet and noise), CI sound quality (“Qualities” section of the SSQ), and CI-related QOL (using the CIQOL) in a group of adult CI users by addressing the following objectives:

1. To define the relationship between CI sound quality ratings and CI-aided speech recognition, with the hypothesis that poorer sound quality ratings correlate with less accurate speech recognition scores due to greater signal degradation.
2. To define the relationship between CI sound quality and CI-related QOL, with the primary hypothesis that poorer sound quality ratings correlate with reduced QOL ratings due to the negative impact of perceiving the degraded auditory signal in everyday life. We also predicted stronger correlations between CI sound quality and the communication, entertainment, and environment domains of the CIQOL compared to other domains, as these domains most directly inquire about the clarity of the auditory signal during speech understanding and music appreciation.
3. To examine how sound quality and speech recognition collectively influence CI-related QOL through regression analyses, with the hypothesis that sound quality will emerge as a significant independent predictor of QOL outcomes when controlling for speech recognition abilities.

## 2. Methods

### 2.1 Study participants

Forty-one postlingually deafened adult CI users with over 1 year of CI experience participated in this study. All participants consented by the protocol approved by the local Institutional Review Board (No. 230093) at Vanderbilt University Medical Center. The sample included 29 males and 12 females, with ages ranging from 18 to 80 years (mean age = 57.4 years). Participants used various listening configurations, including 21 bilateral CI users, 14 bimodal users (CI with a contralateral hearing aid), and 6 unilateral CI users. The sample included devices from three manufacturers: Cochlear (n=22), Advanced Bionics (n=18), and MED-EL (n=1). All participants were native English speakers and were tested in their “best-aided” condition using their everyday listening configuration. See the supplementary material for detailed participant demographics, including individual participant characteristics, listening configurations, and device information (supplementary material Table 1).

### 2.2 Materials and procedures

CI users completed the experiment remotely on the Gorilla Experiment Builder platform (Gorilla Experiment Builder, Cauldron Science Ltd, Cambridge, UK).^19^ They listened to stimuli presented free-field from a single speaker in front of them. All participants completed testing remotely using their personal computers. To standardize the listening level as much as possible across participants, each session began with a sound check that used a reference sentence. Participants were instructed to adjust their volume or processor settings until the sentence sounded both clearly intelligible and at a comfortable listening level. They were then instructed to keep these settings fixed throughout the session. Because participants used their own computers, we could not directly calibrate the sound intensity across users. However, we note that this procedure is consistent with remote testing protocols used in prior CI research^20,21^ and was designed to replicate typical listening conditions for each participant. All speech recognition tasks reported in this study were completed within a single session to avoid variability in device settings across sessions. Importantly, remote testing enabled us to recruit a more diverse group of participants, including individuals from a broader geographic area who may not have been able to travel for in-person testing.

### 2.3 Speech recognition

Participants completed open-set word and sentence recognition tasks in quiet before the noise conditions. Word stimuli consisted of monosyllabic consonant-vowel-consonant (CVC) words from the Multi-talker Corpus of Foreign-Accented English and in-house recordings,^22^ including both lexically easy and hard words. Foreign-accented English was included to increase the perceptual challenge and better represent real-world listening conditions that cochlear implant users encounter, as accented speech places additional demands on the auditory system and may be more sensitive to individual differences in sound quality perception. Lexically easy words had high word frequency (mean log contextual diversity = 3.3) and low neighborhood density (mean = 12.6 neighbors). In contrast, lexically hard words had low word frequency (mean = 1.6) and high neighborhood density (mean = 25.7 neighbors).^23–26^

The word recognition task comprised three blocks: (1) a single female native talker (20 words), (2) four native talkers (five words each), and (3) four non-native talkers (five words each). All blocks included equal numbers of lexically easy and hard words. Sentence materials were from the Speech Perception in Noise (SPIN) Test, produced by four non-native talkers from the same corpus and four native talkers. The sentence recognition task followed the same block structure as the word task: (1) a single female native talker (20 sentences), (2) four native talkers (five sentences each), and (3) four non-native talkers (five sentences each). Sentences were either high-context, low-context, or anomalous (e.g., “the turn twisted the cards”). For both quiet tasks, participants heard a stimulus and repeated it aloud. The tasks were self-paced.

#### 2.3.1 Speech recognition in noise

Participants completed adaptive speech recognition tasks to derive speech recognition thresholds (SRTs) for words and sentences in noise. For word recognition, 60 monosyllabic words from the Multi-talker Corpus were spoken by the same female native talker used in the quiet tasks. Words were combined with six-talker babble that began 500 ms before word onset and ended 250 ms after word offset. On each trial, participants typed the word they heard into a text box. A one–up, one–down adaptive staircase procedure in 1 dB SNR increments was used to determine the 50% correct threshold (SRT-50). The first trial presented a word without background noise, with potential SNRs ranging from +18 to +3 dB. The adaptive procedure varied the noise level while maintaining a constant speech signal level to achieve the target SNR. All participants completed all 60 words, and the SRT-50 was calculated as the average SNR of the final six reversals.

For sentence recognition, 50 sentences from the Veteran’s Affairs Sentence Test (VAST)^27^ were spoken by the same female talker, including 25 high-predictability and 25 low-predictability sentences. Sentences were combined with restaurant noise from the ARTE database [Ambisonic Recordings of Typical Environments (ARTE) Database, Australian Hearing Hub, Macquarie University, Sydney, New South Wales, Australia].^28^ Participants typed only the final word they heard. The adaptive procedure matched the word recognition task, except the SNR range extended from +18 to −1 dB. The SRT-50 was calculated as the average SNR of the final six reversals.

#### 2.3.2 Scoring

Audio responses for quiet tasks were recorded and scored offline. A word response was correct when participants accurately repeated the entire word. Sentence responses were scored for the final word correct. Scores were calculated as averages across all talkers and conditions. Some participants did not complete all tasks (seven missing word recognition in quiet, six missing word recognition in noise, three missing sentence recognition in quiet, five missing sentence recognition in noise) and were excluded from the corresponding analyses.

#### 2.3.3 Questionnaires

Participants completed two validated questionnaires to assess self-reported sound quality and CI-related QOL: the Speech, Spatial and Qualities (SSQ-49) questionnaire^14^ and the Cochlear Implant Quality of Life (CIQOL) questionnaire (Cochlear Limited, Sydney, New South Wales, Australia).^29^ The SSQ is a comprehensive measure of hearing disability across multiple domains, consisting of 49 items. The Qualities section, which was used in this study, evaluates the perceived naturalness and clarity of sounds, the ability to segregate sounds, and listening effort. Items are rated on a scale from 0 (complete disability) to 10 (complete ability). The Qualities section of the SSQ contains 18 items assessing sound segregation, identification of voices and musical pieces, clarity, naturalness, and listening effort. The CIQOL-35 is a validated instrument specifically designed for CI users that measures QOL across six domains: communication, emotional, entertainment, environment, listening effort, and social. The questionnaire comprises 35 items rated on a five-point scale (Never, Rarely, Sometimes, Often, or Always). Global and domain scores are calculated, with higher scores indicating better QOL. There were no missing questionnaire data.

#### 2.3.4 Statistical analysis

All analyses were conducted in *R* version 4.2.0.28 (2025) (R Foundation for Statistical Computing, Vienna, Austria).^30^ Missing data from participants who did not complete all speech recognition tasks were handled using multiple imputation with the mice package, generating 20 imputed datasets with 50 iterations each. Parametric tests were used as all variables demonstrated normal distribution (all *p* > 0.05) using the D’Agostino–Pearson Omnibus K2 test. Our first objective used Pearson correlation coefficients (*α* = 0.05) to examine the relationships between SSQ Qualities scores and speech recognition measures (word and sentence recognition in quiet and noise conditions) using pooled estimates across the imputed datasets. Correlations were calculated by transforming individual correlation coefficients to Fisher’s *z*-scores, averaging these scores across imputations, and then back-transforming to correlation coefficients. Our second objective used the same pooled correlation approach to examine relationships between SSQ Qualities scores and CIQOL scores, including global and domain scores (communication, emotional, entertainment, environment, listening effort, and social). Our third objective used linear regression analyses to examine how sound quality and speech recognition collectively predict CI-related QOL outcomes. Separate regression models were fit for CIQOL global scores and each domain score. SSQ Qualities scores and all four speech recognition measures (words and sentences in quiet and noise conditions, pooled across talker conditions) were simultaneous predictors without interaction terms. This approach allows us to examine their independent contributions to QOL while accounting for any shared variance between these predictors. Although we hypothesized relationships between sound quality and speech recognition measures, including both factors in the same models, enabled us to determine which factor is more strongly associated with CI-related QOL outcomes when controlling for the other. Results were pooled across imputed datasets, and standardized coefficients (*β*) were calculated to assess effect sizes. *R*-squared values were computed for each model to determine the proportion of variance explained. The Benjamini–Hochberg procedure was used to control for multiple comparisons across domain-specific analyses. Visual representations of relationships between variables were created using the ggplot2 package (Posit PBC, Boston, MA).

## 3. Results

### 3.1 The relationship between CI sound quality and CI-aided speech recognition

**Fig. 1.**
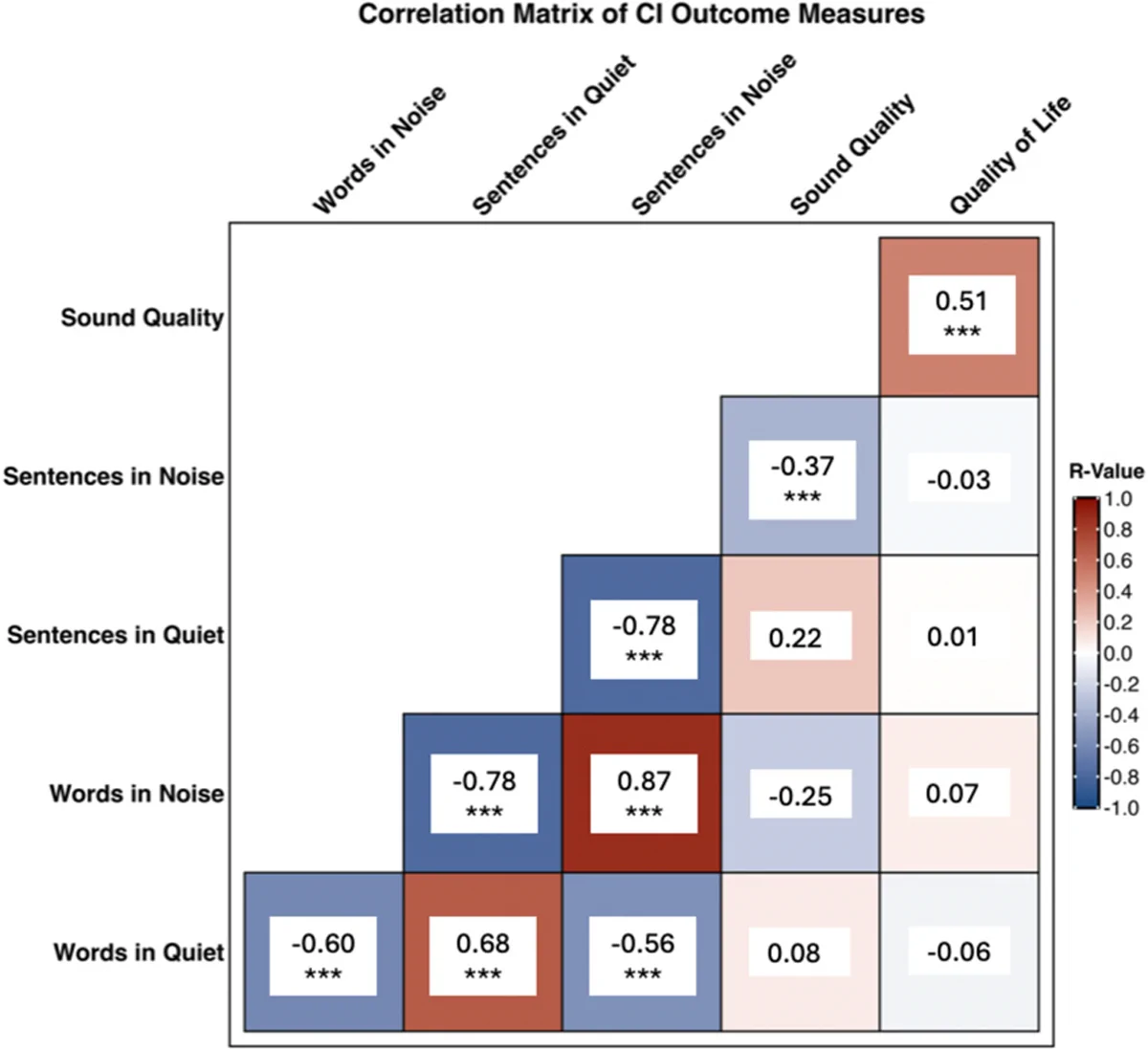
Pearson correlation matrix showing the relationships between speech recognition measures (Words in Quiet, Words in Noise, Sentences in Quiet, and Sentences in Noise); SSQ Qualities section scores (labeled as Sound Quality), and CIQOL-35 global scores (labeled as Quality of Life). For speech in quiet measures and questionnaires, higher scores indicate better performance, whereas for speech-in-noise measures, lower scores indicate better performance. Red colors indicate positive relationships and blue colors indicate negative relationships. Color intensity represents relationship strength. Significance level is denoted by asterisks (*p < 0.05; **p < 0.01, ***p < 0.001).

SSQ Qualities scores showed varying relationships with speech recognition measures (Fig. 1). For speech recognition assessments, performance is measured differently across tasks: in quiet tasks, higher percentage scores indicate better performance; in noise tasks, lower thresholds (dB SNR) represent better performance as they demonstrate the ability to understand speech at more challenging signal-to-noise ratios (SNRs). Similarly, on the SSQ Qualities section, higher numerical ratings (on a scale of 0–10) reflect better perceived sound quality. For example, sentences in noise showed a weak negative correlation with sound quality ratings (*r* = −0.37, *p* < 0.001), indicating that better sound quality ratings were associated with better sentence recognition in noise. However, none of the other speech recognition measures were significantly correlated with sound quality ratings (*r* values = −0.25–0.22, *p* values > 0.05).

### 3.2 The relationship between CI sound quality and CI-related QOL

**Fig. 2.**
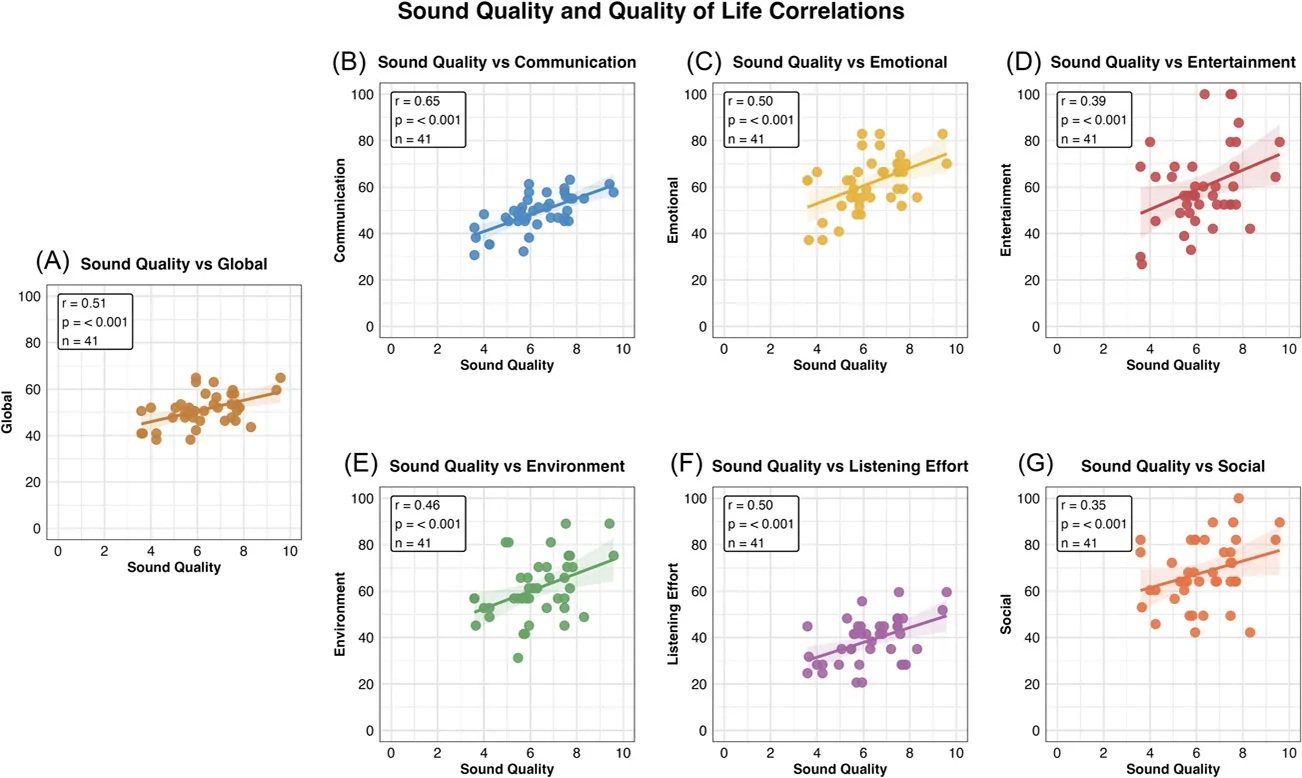
Scatterplots showing relationships between SSQ Qualities scores and CIQOL domains. (A) Global scores show the overall relationship between sound quality and quality of life. (B)–(G) Domain-specific relationships: Communication, emotional, entertainment, environment, listening effort, and social. Regression lines (colored by domain) with 95% confidence intervals are shown. Results are based on pooled estimates from multiple imputation analyses.

SSQ Qualities scores showed significant positive correlations with CIQOL global scores (*r* = 0.51, *p* < 0.001) and all domain scores (*r* values = 0.35–0.65, *p* values < 0.001), as shown in Fig. 2. Higher sound quality ratings were associated with better QOL outcomes. The strongest correlations with sound quality were observed in the communication domain (*r* = 0.65, *p* < 0.001), followed by the listening effort and emotional domains (both *r* = 0.50, *p* < 0.001), then the environment domain (*r* = 0.46, *p* < 0.001), while the entertainment (*r* = 0.39, *p* < 0.001) and social domains (*r* = 0.35, *p* < 0.001) demonstrated relatively weaker, though still significant, correlations.

### 3.3 Direct and indirect effects of CI sound quality and speech recognition on CI-related QOL

**Fig. 3.**
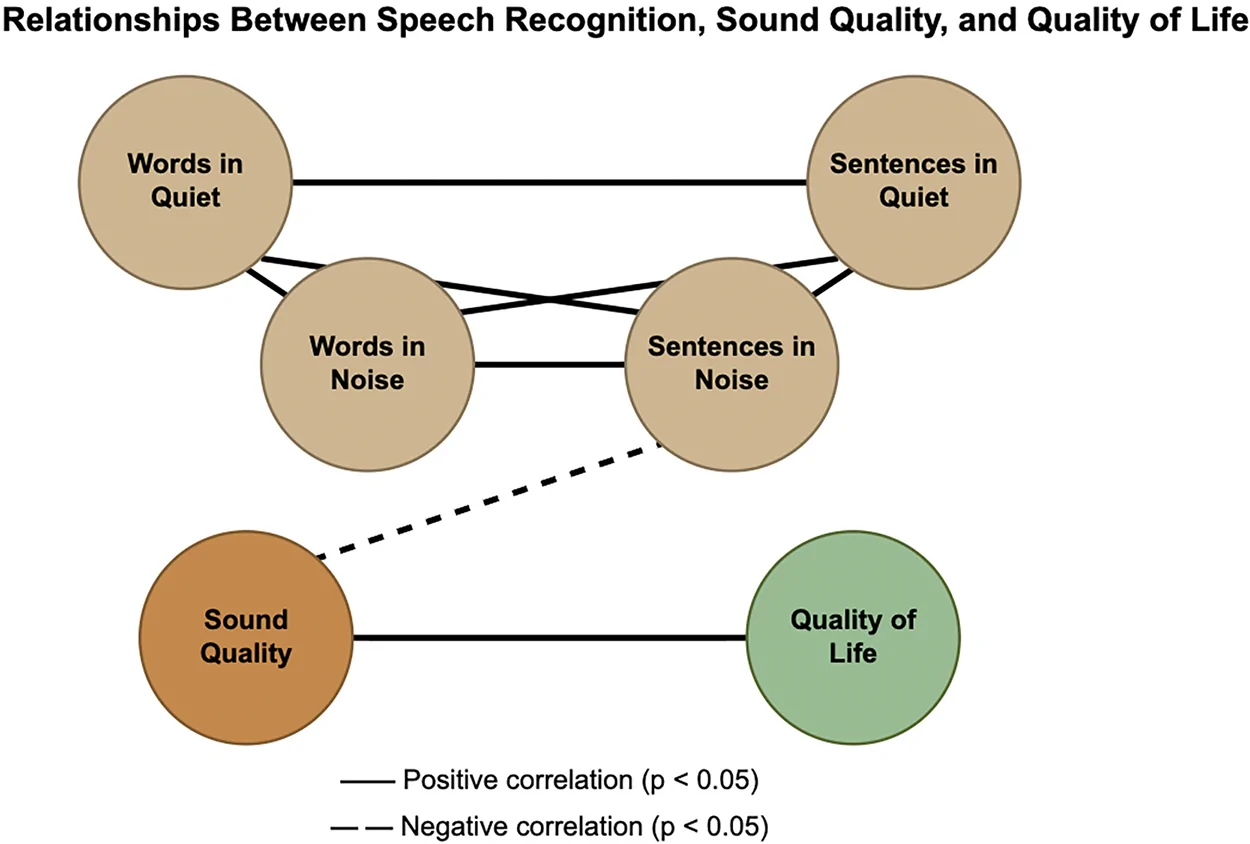
Path diagram depicting relationships between sound quality, speech recognition, and quality of life in adult CI users. Red lines indicate positive relationships and blue lines indicate negative relationships. Line thickness and color intensity represent relationship strength, with thicker/darker arrows indicating stronger relationships. Solid lines indicate significant relationships and dashed lines represent non-significant relationships. The negative correlations observed between some speech recognition measures likely reflect the inherent variability in performance across different listening conditions and test materials.

Linear regression analyses revealed significant relationships between CI sound quality and CI-related QOL outcomes, as shown in the path diagram (Fig. 3). Sound quality emerged as the only significant predictor of CIQOL Global scores, with each one-point increase in SSQ Qualities scores associated with a 2.67-point increase in CIQOL Global scores. Domain-specific analyses showed that sound quality significantly predicted scores across all domains: communication, social, environment, listening effort, emotional, and entertainment domains (Table 1). Speech recognition measures were not significant predictors for any of the domains (*p* values > 0.05). These findings indicate that better CI sound quality, rather than speech understanding ability, is the key driver of improved CI-related QOL across nearly all domains, explaining between 17% and 50% of the variation in scores.

### 3.4 Consistency of relationships across listening configurations

To examine whether the heterogeneous CI configurations in our sample affected the primary findings, we analyzed the relationship between sound quality and CIQOL global scores within each subgroup. Despite different sample sizes across configurations, *post hoc* Pearson correlations revealed consistent positive relationships: bilateral CI users (*r* = 0.49, *p* = 0.035, *n* = 21), bimodal users (*r* = 0.69, *p* = 0.019, *n* = 14), and unilateral CI users (*r* = 0.31, *p* = 0.544, *n* = 6). Fisher’s *z*-tests comparing correlation coefficients showed no significant differences between any pair of configurations (bilateral vs bimodal: *z* = −0.73, *p* = 0.465; bilateral vs unilateral: *z* = 0.33, *p* = 0.744; bimodal vs unilateral: *z* = 0.77, *p* = 0.441), indicating that the sound quality-QOL relationship is consistent across different CI device configurations.

## 4. Discussion

This study investigated relationships between CI sound quality, speech recognition, and QOL in adult CI users. Our findings reveal that sound quality, rather than speech recognition performance, serves as the primary predictor of CI-related QOL outcomes across multiple domains.

### 4.1 Sound quality and speech recognition relationships

The relationship between sound quality ratings and speech recognition was more nuanced than initially hypothesized. While we expected poorer sound quality to correlate with reduced speech recognition across all conditions due to signal degradation, this relationship was only observed for speech recognition in noise. This disconnect between how well CI users understand speech and how they judge the sound quality aligns with the broader literature on speech clarity and listening effort.^31^ Previous research has shown that perceived speech clarity does not necessarily correlate with speech recognition performance, as these measures reflect distinct cognitive and perceptual processes.^32,33^ While speech recognition relies heavily on pattern matching and top–down linguistic processes that may compensate for degraded acoustic input,^7^ sound quality perception may be more directly tied to bottom–up processing of the acoustic signal itself. Studies examining clarity ratings have demonstrated that while these ratings generally decrease as signal degradation increases, this relationship is not strictly linear or proportional across all degradation levels,^32,34^ suggesting complex interactions between top–down and bottom–up processes.^7^ This non-linear relationship may explain why CI users’ subjective sound quality ratings diverged from their speech recognition scores, particularly in quiet conditions where top–down compensation might be more effective.^3^

Our findings are consistent with previous research examining the relationships between the SSQ and speech recognition. For example, Capretta and Moberly found weak correlations between overall SSQ scores and word recognition in quiet and negligible correlations with sentence recognition in quiet, while correlations were somewhat stronger for the Speech section of the SSQ.^16^ Similarly, Fuller and colleagues found moderate correlations between overall SSQ scores and word recognition, with similar relationships across all three sections.^17^ Our results showing weak correlations between sound quality and most speech measures support these prior findings and confirm that sound quality captures distinct aspects of the CI experience beyond speech recognition performance. The selective correlation we observed with speech-in-noise performance suggests that sound quality perception may be particularly relevant in challenging listening conditions where the SNR is poor and top–down compensation is more limited. This finding aligns with the fundamental challenges of CI signal processing, where channel interaction and limited spectral resolution become especially problematic in complex acoustic environments.^11,12^ Moreover, speech-in-noise more closely represents real-world listening conditions and better predicts real-world communication difficulties than speech-in-quiet, as everyday communication typically occurs in the presence of competing sounds and acoustic challenges.^35–37^ Therefore, our finding that sound quality ratings correlate specifically with speech-in-noise performance, rather than speech-in-quiet, suggests that sound quality may be particularly important for predicting CI users’ real-world communication experiences.

### 4.2 Sound quality and QOL relationships

The robust correlations between CI sound quality and QOL measures support our second hypothesis that sound quality impacts overall CI-related QOL. Our finding that speech recognition measures did not predict QOL outcomes is consistent with a recent meta-analysis by McRackan and colleagues, showing weak correlations between speech recognition and QOL.^18^ This suggests that traditional speech measures capture only a small portion of what drives patient-reported outcomes, highlighting the importance of incorporating sound quality measures into clinical assessments.

The strongest correlation was observed with the communication domain, suggesting that the perceived clarity and naturalness of sounds significantly impact daily communication experiences, potentially by reducing the cognitive resources required for speech understanding and making communication less effortful and more enjoyable. This relationship is further supported by examining the specific content of the CIQOL communication domain, which includes multiple items addressing performance in challenging listening environments (e.g., multi-talker conversations, crowded environments, noisy places) and questions about voice naturalness. The strong relationship between this communication domain and sound quality ratings likely reflects the fundamental role of signal fidelity in these scenarios, as understanding in more complex acoustic environments more heavily depends on spectral resolution.^38–40^

This pattern is particularly noteworthy when examining the content overlap between the SSQ Qualities section and CIQOL domains. The SSQ Qualities section includes items specifically assessing naturalness and clarity of sounds (e.g., “Do everyday sounds seem clear to you?” and “Do other people’s voices sound clear and natural?”), which are mirrored in the CIQOL communication domain (e.g., “Other people’s voices sound clear and natural to me”). Similarly, both questionnaires address listening effort, with the SSQ asking about concentration requirements and the CIQOL including specific items about effort in various listening situations. The strong correlation between the environment domain of the CIQOL and the SSQ Qualities section likely reflects the shared focus on everyday sound perception, with both instruments asking about environmental sound clarity and recognition. Interestingly, while emotional showed significant correlations with sound quality, the entertainment domain demonstrated the weakest relationship and demonstrated evidence of a ceiling effect. The weaker relationship observed in the entertainment domain may be partially explained by the unique challenges that CIs present for music, which is a significant component of this domain. Studies have consistently shown that music perception and enjoyment remain particularly problematic for CI users.^10,41^ Importantly, music enjoyment is largely independent of both music and speech perception, with weak to nonexistent correlations between behavioral perceptual measures and subjective music appraisal ratings,^42–44^ which supports our finding that sound quality ratings—rather than speech recognition measures—were more predictive of entertainment-related QOL outcomes.

### 4.3 Clinical implications and future directions

The regression analyses revealed that sound quality independently predicts QOL outcomes across multiple domains, with substantial effect sizes suggesting that even modest improvements in sound quality could yield meaningful benefits for CI users’ daily lives. The lack of direct effects from speech recognition measures on QOL, combined with the strong predictive power of the sound quality challenges the traditional clinical focus on speech recognition as the primary metric of CI success.

These findings have important clinical implications. First, they suggest that traditional speech recognition measures alone may not capture aspects of the CI that significantly impact the user’s daily experiences. Second, they highlight the potential value of incorporating sound quality measures into clinical assessments. Finally, they suggest that interventions to improve CI outcomes should consider sound quality optimization alongside speech recognition, as better sound quality may enhance QOL even if clinical speech recognition scores remain unchanged. Our findings were also consistent across listening configurations, suggesting that improving CI sound quality is important to all CI users regardless of their listening configuration.

Future research should pursue several complementary directions to advance our understanding of CI sound quality. First, studies should investigate whether specific dimensions of sound quality (e.g., naturalness, clarity, or pleasantness) differentially impact QOL domains, which could inform targeted interventions. Second, systematic examination of how device characteristics—including CI manufacturer, electrode type and length, insertion depth, and mapping parameters—influence sound quality perception is critically needed. Third, qualitative studies are needed to understand how CI users conceptualize and experience sound quality in their daily lives, which could inform the development of more comprehensive, clinically feasible assessment tools. Finally, longitudinal studies could examine how sound quality-QOL relationships evolve over time, whether early sound quality experiences predict long-term outcomes, and how interventions targeting sound quality impact both immediate and sustained QOL improvements.

### 4.4 Limitations

Several limitations of this study should be noted. First, sound quality was assessed using only the SSQ Qualities section. Future studies might benefit from developing and validating CI-specific sound quality measures. Second, the cross sectional nature of this study prevents causal inferences about the relationships between sound quality and QOL. Third, while our sample size was adequate for detecting moderate to strong relationships, larger samples might reveal more subtle connections, particularly within specific QOL domains. Fourth, our remote testing approach, while enabling broader participant recruitment, introduced potential environmental variability. Although participants were instructed to complete tasks in quiet rooms, ambient noise and other environmental factors may have varied across participants. Additionally, our use of typed responses for speech recognition tasks may have favored participants more comfortable with keyboard use or digital interfaces, potentially introducing bias related to digital literacy. However, the strong correlations observed between different speech recognition measures suggest that response format did not substantially alter individual performance patterns. We mitigated some remote testing concerns by completing all speech recognition tasks within a single session using standardized sound check procedures.^20,21^ Finally, our heterogeneous sample introduced variability through various listening configurations, and our small unilateral CI subgroup limited the precision of subgroup analyses. We were unable to collect detailed CI device and programming information that could impact both sound quality and speech recognition outcomes. This dataset was originally collected for a different research question with participants recruited nationally rather than from a single CI center, limiting our access to comprehensive medical records. Although these device-related factors may have influenced absolute sound quality or speech recognition scores, we would not necessarily expect them to alter the strength or direction of the observed relationship between perceived sound quality and QOL. Additionally, our sample was not adequately powered to detect meaningful differences between device manufacturers, which could have impacted our results. However, this assumption should be confirmed in future studies specifically designed to examine the effects of device characteristics on sound quality and its relationship to QOL.

## 5. Summary

This study demonstrates that CI sound quality has a unique and substantial relationship with QOL independent of speech recognition abilities in adult CI users. While speech recognition in noise showed a weak negative correlation with sound quality ratings, sound quality demonstrated robust positive correlations with QOL across multiple domains, explaining substantial score variability. Regression analyses revealed that improvements in sound quality were associated with substantial gains in QOL scores, with particularly strong effects in the communication, social, and environment domains. However, the entertainment domain showed the weakest relationship and evidence of a ceiling effect, suggesting diminishing returns beyond certain sound quality thresholds.

These findings indicate that speech recognition alone does not adequately capture crucial aspects of CI users’ daily experiences. Moving forward, sound quality should be considered as a distinct and important outcome measure. Better sound quality, rather than speech understanding ability, is the primary factor driving CI-related QOL improvements. Given these results, future research should focus on developing comprehensive CI-specific sound quality assessments while examining how these relationships evolve longitudinally. Further, evidence-based interventions to improve CI sound quality should be identified to enhance users’ everyday experiences, particularly in challenging listening environments where sound quality appears critical for performance and satisfaction.

## Supplementary Material

Supplementary Material

Supplementary Material

See the supplementary material for participant demographics.

## Figures and Tables

**Table 1.** Sound quality as a predictor of CIQOL scores.

CIQOL domain	*R* ^2^	*β*	*p*

Global	0.32	2.67	0.002
Communication	0.50	4.06	<0.001
Social	0.39	4.58	0.005
Environment	0.32	4.60	0.005
Listening effort	0.31	3.87	0.002
Emotional	0.30	4.18	0.004
Entertainment	0.17	4.67	0.046

## Data Availability

The data that support the findings of this study are available from the corresponding author upon reasonable request. The data are not publicly available due to containing information that could compromise research participant privacy.
