# Supplementary material for: Sound quality, not speech recognition, explains cochlear implant-related quality of life outcomes
Source: JASA Express Lett. Author manuscript; Available in PMC 2026 Aug 21. (PMC13495099; doi:10.1121/10.0039069)
Supplement: Supplementary file 1 [file NIHMS2152597-supplement-Supplementary_Material.docx]

Supplemental Table 1. Participant Demographics

| Participant | Age | Sex | Listening configuration | CI manufacturer | Age at CI | CI |
| --- | --- | --- | --- | --- | --- | --- |
| 1 | 73 | Male | Bilateral | Cochlear | 65 (R), 66 (L) | Both |
| 2 | 70 | Male | Bimodal | Cochlear | 61 | Right |
| 3 | 72 | Male | Bilateral | Cochlear | 55 (R), 56 (L) | Both |
| 4 | 59 | Male | Bilateral | Cochlear | 58 (R), 59 (L) | Both |
| 5 | 54 | Female | Bilateral | Cochlear | 51 (R), 53 (L) | Both |
| 6 | 77 | Female | Unilateral | Cochlear | 71 | Left |
| 7 | 80 | Male | Bimodal | Cochlear | 78 | Left |
| 8 | 51 | Male | Unilateral | Cochlear | 49 | Right |
| 9 | 60 | Female | Bilateral | Cochlear | 51 (R), 52 (L) | Both |
| 10 | 58 | Female | Bimodal | Cochlear | 55 | Right |
| 11 | 18 | Male | Unilateral | Cochlear | 17 | Left |
| 12 | 49 | Female | Bimodal | Cochlear | 46 | Left |
| 13 | 61 | Male | Bimodal | Cochlear | 58 | Left |
| 14 | 52 | Male | Unilateral | Cochlear | 48 | Right |
| 15 | 68 | Male | Bilateral | Advanced Bionics | 65 (R), 66 (L) | Both |
| 16 | 49 | Male | Bilateral | Advanced Bionics | 45 (R), 46 (L) | Both |
| 17 | 66 | Female | Bilateral | Advanced Bionics | 63 (R), 64 (L) | Both |
| 18 | 55 | Male | Bilateral | Advanced Bionics | 51 (R), 53 (L) | Both |
| 19 | 58 | Male | Bilateral | Cochlear | 56 (R), 57 (L) | Both |
| 20 | 55 | Male | Bimodal | Cochlear | 55 | Right |
| 21 | 50 | Male | Unilateral | Advanced Bionics | 46 | Right |
| 22 | 51 | Female | Bilateral | Advanced Bionics | 47 (R), 49 (L) | Both |
| 23 | 60 | Male | Bimodal | Cochlear | 58 | Left |
| 24 | 60 | Male | Bimodal | Advanced Bionics | 57 | Right |
| 25 | 64 | Male | Bilateral | Advanced Bionics | 60 (R), 62 (L) | Both |
| 26 | 34 | Male | Bilateral | Cochlear | 30 (R), 32 (L) | Both |
| 27 | 59 | Female | Bilateral | MED-EL | 55 (R), 57 (L) | Both |
| 28 | 67 | Female | Bilateral | Cochlear | 64 (R), 65 (L) | Both |
| 29 | 66 | Male | Bimodal | Cochlear | 64 | Left |
| 30 | 61 | Male | Bimodal | Cochlear | 57 | Right |
| 31 | 56 | Male | Bilateral | Advanced Bionics | 52 (R), 54 (L) | Both |
| 32 | 57 | Male | Unilateral | Cochlear | 55 | Left |
| 33 | 58 | Male | Bimodal | Advanced Bionics | 54 | Right |
| 34 | 59 | Female | Bilateral | Advanced Bionics | 55 (R), 57 (L) | Both |
| 35 | 52 | Male | Bimodal | Advanced Bionics | 48 | Right |
| 36 | 53 | Male | Bilateral | Advanced Bionics | 49 (R), 51 (L) | Both |
| 37 | 54 | Female | Bimodal | Advanced Bionics | 52 | Left |
| 38 | 56 | Female | Bilateral | Cochlear | 52 (R), 54 (L) | Both |
| 39 | 57 | Male | Bilateral | Cochlear | 53 (R), 55 (L) | Both |
| 40 | 59 | Male | Bilateral | Advanced Bionics | 55 (R), 57 (L) | Both |
| 41 | 62 | Male | Bimodal | Cochlear | 58 | Right |
